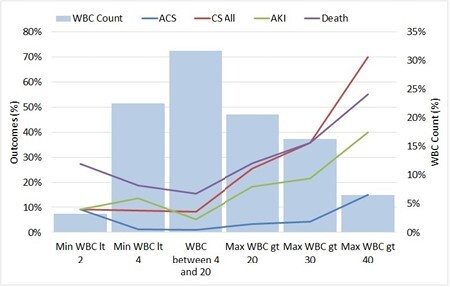# 507 Decrease in Total Leukocyte Count Is Associated with Acute Kidney Injury After Severe Burn

**DOI:** 10.1093/jbcr/irae036.142

**Published:** 2024-04-17

**Authors:** James K Aden, Julie A Rizzo, Steven G Schauer, Tam N Pham, Jose Salinas, Laura S Johnson, Rachel Harris

**Affiliations:** U. S. Army Graduate Medical Education, Ft. Sam Houston, TX; Brooke Army Medical Center, Houston, TX; University of Colorado School of Medicine, Aurora, CO; University of Washington, Harborview Burn Centre, Seattle, WA; U.S. Army Institute of Surgical Research, Ft Sam Houston, TX; Walter L Ingram Burn Center / Emory at Grady, Atlanta, GA; Brooke Army Medical Center, San Antonio, TX; U. S. Army Graduate Medical Education, Ft. Sam Houston, TX; Brooke Army Medical Center, Houston, TX; University of Colorado School of Medicine, Aurora, CO; University of Washington, Harborview Burn Centre, Seattle, WA; U.S. Army Institute of Surgical Research, Ft Sam Houston, TX; Walter L Ingram Burn Center / Emory at Grady, Atlanta, GA; Brooke Army Medical Center, San Antonio, TX; U. S. Army Graduate Medical Education, Ft. Sam Houston, TX; Brooke Army Medical Center, Houston, TX; University of Colorado School of Medicine, Aurora, CO; University of Washington, Harborview Burn Centre, Seattle, WA; U.S. Army Institute of Surgical Research, Ft Sam Houston, TX; Walter L Ingram Burn Center / Emory at Grady, Atlanta, GA; Brooke Army Medical Center, San Antonio, TX; U. S. Army Graduate Medical Education, Ft. Sam Houston, TX; Brooke Army Medical Center, Houston, TX; University of Colorado School of Medicine, Aurora, CO; University of Washington, Harborview Burn Centre, Seattle, WA; U.S. Army Institute of Surgical Research, Ft Sam Houston, TX; Walter L Ingram Burn Center / Emory at Grady, Atlanta, GA; Brooke Army Medical Center, San Antonio, TX; U. S. Army Graduate Medical Education, Ft. Sam Houston, TX; Brooke Army Medical Center, Houston, TX; University of Colorado School of Medicine, Aurora, CO; University of Washington, Harborview Burn Centre, Seattle, WA; U.S. Army Institute of Surgical Research, Ft Sam Houston, TX; Walter L Ingram Burn Center / Emory at Grady, Atlanta, GA; Brooke Army Medical Center, San Antonio, TX; U. S. Army Graduate Medical Education, Ft. Sam Houston, TX; Brooke Army Medical Center, Houston, TX; University of Colorado School of Medicine, Aurora, CO; University of Washington, Harborview Burn Centre, Seattle, WA; U.S. Army Institute of Surgical Research, Ft Sam Houston, TX; Walter L Ingram Burn Center / Emory at Grady, Atlanta, GA; Brooke Army Medical Center, San Antonio, TX; U. S. Army Graduate Medical Education, Ft. Sam Houston, TX; Brooke Army Medical Center, Houston, TX; University of Colorado School of Medicine, Aurora, CO; University of Washington, Harborview Burn Centre, Seattle, WA; U.S. Army Institute of Surgical Research, Ft Sam Houston, TX; Walter L Ingram Burn Center / Emory at Grady, Atlanta, GA; Brooke Army Medical Center, San Antonio, TX

## Abstract

**Introduction:**

Whereas leukocytosis is common immediately after major burn injury, total leukocyte count (TLC) often decreases 72-96 hours post-injury. It is reported that up to 50% of patients may experience leukopenia during this timeframe, which is typically self-limiting. The incidence of early (< 72 hours) leukopenia and its implications have not been well-described. This analysis sought to determine if early and extreme decreases in TLC after burn injury was associated with increased fluid requirements or resuscitation-related complications in patients after burn injury.

**Methods:**

The Burn Navigator (BN) database is composed of patients with > 20% TBSA, > 40kg that were resuscitated utilizing the BN. Patients were split into study arms based on decreases in TLC relative to admission value within the first 24 hours after injury. Demographics were collected on the two study arms as well as resuscitation volumes, resuscitation-related complications and 7-day survival.

**Results:**

A total of 295 patients were included in the analysis. Demographics were similar between the two groups. Patients with a larger decrease in TLC from admission had more full-thickness burns (14.2 [2,36]) and greater overall TBSA (41 [30.2, 57.4]) than those with a smaller decrease (7.7 [0.3, 14.8], 7.5 [23, 36], both p < 0.001). Patients with a decrease in TLC from admission greater than 15 points had a higher incidence of admission myoglobinuria (55.6%) than those with a TLC decrease less than 15 (44.4 %, p = 0.01). Patients with a greater TLC drop had an increased incidence of developing acute kidney injury (AKI) in the first 48 hours of resuscitation (62.9% vs. 37.1%, p = 0.003) despite receiving more fluid. After controlling for TBSA, patients with a greater TLC drop still had a higher incidence of AKI. Patients with a TLC drop greater than 15 had significantly decreased 7-day survival (66.7% lived) compared to those with a TLC drop of less than 15 (83.7% lived, p< 0.001).

**Conclusions:**

Acute leukopenia alone was not associated with mortality after thermal injury. An early, severe decrease in TLC was found to be associated with early AKI and this may be readily identified by admission myoglobinuria. Future studies need to investigate the mechanism behind this association and possible early therapeutic interventions to prevent AKI and decrease mortality after severe burn injury.

**Applicability of Research to Practice:**

Variability in leukocyte response to thermal injury may help guide both clinician optimization of organ function, and allow for realistic goals of care to be discussed with patient advocates.